# Analysis of Insurance Type and Emergency Severity Index (ESI) Level in an Emergency Department Population: A Single-Center Prospective Observational Study

**DOI:** 10.7759/cureus.114934

**Published:** 2026-08-21

**Authors:** Lawrence Goriel, Matthew Boutorwick

**Affiliations:** 1 Emergency Medicine, Henry Ford Health, Warren, USA

**Keywords:** emergency severity index, insurance, medicaid, uninsured, utilization

## Abstract

Background

Health insurance disparities are frequently cited as contributors to inefficiencies and rising costs in the United States healthcare system. While uninsured patients have historically been assumed to delay care and present with higher acuity, the increasing prevalence of government-sponsored insurance raises questions about emergency department utilization patterns across insurance groups.

Objective

The objective of this study is to evaluate the association between insurance type (private, government-sponsored, and uninsured) and Emergency Severity Index (ESI) level among emergency department patients.

Methods

This single-day prospective observational study was conducted at Henry Ford Warren Emergency Department on December 1, 2025. An a priori power calculation determined that 167 patients were required for 80% power to detect a medium effect size (Cohen’s w = 0.30, α = 0.05, df = 8). All patients assigned an ESI level (1-5) with documented insurance information were included. Insurance was categorized as private, government-sponsored, or uninsured. Descriptive statistics were calculated, and a Pearson chi-square test of independence was used to assess the association between insurance type and ESI level; Cramer’s V was calculated as a measure of effect size.

Results

A total of 203 patient encounters met the inclusion criteria: 152 (74.9%) government-sponsored, 35 (17.2%) private, and 16 (7.9%) uninsured. ESI level 3 represented the most common triage category overall (107/203, 52.7%). ESI level 3 accounted for 69 of 152 government-sponsored patients (45.4%), 27 of 35 privately insured patients (77.1%), and 11 of 16 uninsured patients (68.8%). Pearson’s chi-square analysis demonstrated no statistically significant association between insurance type and ESI level (χ²(8) = 14.27, p = 0.075; p < 0.05 was required to determine statistical significance). Cramer’s V was 0.187, indicating a small effect size.

Conclusions

No statistically significant association was identified between insurance type and emergency department acuity, as measured by ESI level, in this single-center, single-day sample. These preliminary findings do not support insurance status alone as a predictor of presentation severity; larger, multicenter studies are needed to confirm this observation.

## Introduction

Rising healthcare expenditures in the United States have been attributed in part to disparities in insurance coverage and access to care [1]. Historically, uninsured patients have been presumed to delay care until disease severity necessitates emergency evaluation, resulting in higher-acuity presentations and increased downstream healthcare costs. Over the past decade, however, enrollment in Medicaid and other government-sponsored insurance programs has expanded substantially, while overall healthcare expenditures have continued to rise [2].

Between 2009 and 2019, Medicaid enrollment among individuals under age 65 increased from 42.4 million to 55.5 million, a 30% increase, while the uninsured population declined from 17.5% to 12.0%. Despite these shifts, national healthcare expenditures increased from $2.5 trillion to $3.8 trillion annually, a 52% increase [3]. These trends raise the question of whether expanded insurance coverage has been associated with any measurable shift in emergency department acuity or utilization patterns.

Emergency department acuity was determined using the Emergency Severity Index (ESI), a validated five-level triage algorithm that categorizes patients according to illness severity and anticipated resource utilization [4,5]. The ESI has demonstrated strong inter-rater reliability and validity across diverse emergency department settings and is the standard triage instrument used at the study institution. The ESI was used solely as a standard clinical assessment tool; no copyrighted ESI materials were reproduced in this manuscript.

This study aimed to evaluate the association between insurance type and emergency department acuity, as measured by ESI level, in a single community emergency department.

## Materials and methods

Study design and setting

This prospective observational study was conducted at Henry Ford Warren Emergency Department, a community-based emergency department in Warren, Michigan, over a single 24-hour study period on December 1, 2025.

Study population

All patients presenting to the emergency department during the study period who were assigned an ESI level (1-5) and had documented insurance information were eligible for inclusion. Insurance information was verified utilizing hospital protocols delegated to registration staff. Registration staff were instructed to verify any questionable insurance status with the patient and the insuring entity. Patients were excluded if they lacked evidence to support claimed insured status, were not assigned an ESI level, represented duplicate registrations, or left without being seen.

Data collection

Data were collected via direct observation and retrospective chart review using a standardized data collection tool. Variables collected included date and time of arrival, medical record number, ESI level, primary insurance, and insurance category. Government-sponsored insurance included both Medicaid and Medicare; these subcategories were recorded but were not part of the prespecified primary analysis (see Statistical Analysis section).

Outcomes

The primary outcome was ESI level at triage: Level 1 (highest priority), Level 2 (high priority), Level 3 (moderate priority), Level 4 (low priority), Level 5 (lowest priority). The primary exposure variable was insurance type (private, government-sponsored, or uninsured).

Statistical analysis

Categorical variables were summarized using frequencies and percentages. The association between insurance type and ESI level was evaluated using the Pearson chi-square test of independence, with Cramer’s V used to quantify effect size. Statistical significance was defined as a two-sided p-value <0.05. A comparison of ESI distribution between Medicare and Medicaid subgroups within the government-sponsored category was conducted as an exploratory, post hoc analysis and was not prespecified; it is reported descriptively in the Results section and should be interpreted as hypothesis-generating only.

An a priori power calculation was performed prior to data collection. For a chi-square test of independence with 8 degrees of freedom (reflecting the planned 3 × 5 insurance-by-ESI contingency table) and α = 0.05, a target effect size of Cohen’s w = 0.30 (medium effect) required a sample size of 167 patients to achieve 80% power and 213 patients to achieve 90% power. Enrollment of 203 patients was targeted to satisfy the 80% power threshold with margin, and this figure was reached within the single-day study period.

A supplemental post hoc power analysis was conducted after data collection to contextualize the null result and characterize which effect sizes the achieved sample could and could not reliably detect. At the enrolled sample size of 203, the study retained 99.4% power to detect a medium effect (w = 0.30), consistent with the a priori calculation, but only 13.2% power to detect a small effect (w = 0.10). Power to detect the effect size actually observed in this study (w = 0.265, equivalent to Cramer’s V = 0.187) was 77.5%, and a sample size of approximately 214 patients would have been required to achieve 80% power for this specific effect - only marginally more than the 203 patients enrolled. Detecting a conventionally small effect (w = 0.10) with 80% power would have required approximately 1,503 patients. Post hoc power estimates based on the observed effect size are mathematically related to the obtained p-value and should be interpreted cautiously; they are presented here to illustrate the range of effect sizes this sample was and was not equipped to detect, rather than as an independent confirmation of the null result.

Ethics statement

This study was reviewed by the Henry Ford Health Research Administration Review Board and deemed exempt, with a waiver of informed consent, as the study utilized previously gathered clinical or administrative datasets originally collected for standard medical diagnosis or treatment.

## Results

A total of 203 patient encounters met inclusion criteria. Insurance distribution was as follows: government-sponsored insurance, 152 patients (74.9%); private insurance, 35 patients (17.2%); uninsured, 16 patients (7.9%).

**Figure 1:**
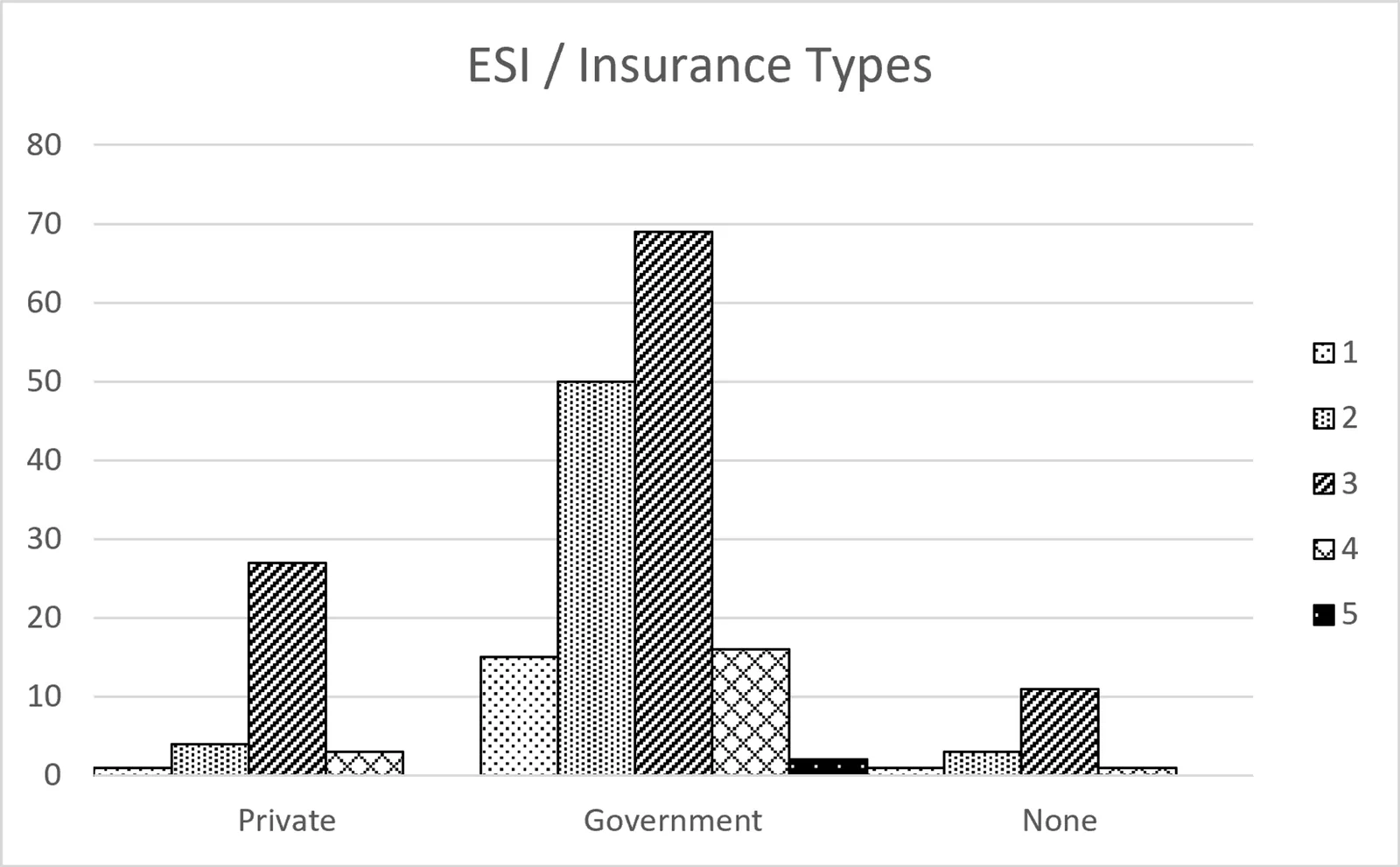
Clustered Column Bar Graph of ESI by Insurance Type ESI: Emergency Severity Index

ESI level 3 was the most common triage category across all insurance groups, accounting for 52.7% of all encounters. Government-sponsored patients accounted for the majority of ESI 1-3 presentations (134/181, 74.0%), consistent with this group also representing the largest share of the overall sample (Figure 1).

In an exploratory, post hoc comparison, Medicare patients within the government-sponsored group demonstrated a numerically higher proportion of ESI 1-2 presentations than Medicaid patients. Because this comparison was not prespecified and subgroup counts were not formally tabulated, these figures are not reported quantitatively here, and the observation should be treated as hypothesis-generating rather than confirmatory.

Pearson’s chi-square test of independence demonstrated no statistically significant association between insurance type and ESI level (χ²(8) = 14.27, p = 0.075). The corresponding Cramer’s V was 0.187, indicating a small effect size (Table 1).

**Table 1:** Distribution of ESI Levels by Insurance Type Data are presented as n (% within insurance category). p-values calculated using the Pearson chi-square test of independence. Test statistics: χ² = 14.27, degrees of freedom (df) = 8, p = 0.075; Cramer’s V = 0.187. Statistical significance was defined as p < 0.05. ESI: Emergency Severity Index

Insurance Type	ESI 1 n (%)	ESI 2 n (%)	ESI 3 n (%)	ESI 4 n (%)	ESI 5 n (%)	Total
Government-sponsored	15 (9.9)	50 (32.9)	69 (45.4)	16 (10.5)	2 (1.3)	152
Private	1 (2.9)	4 (11.4)	27 (77.1)	3 (8.6)	0 (0)	35
Uninsured	1 (6.3)	3 (18.8)	11 (68.8)	1 (6.3)	0 (0)	16
Total	17 (8.4)	57 (28.1)	107 (52.7)	20 (9.9)	2 (1.0)	203 (100)

## Discussion

In this single-center, single-day sample, government-sponsored insurance - predominantly Medicaid - was not associated with lower-acuity emergency department utilization as measured by ESI level. Despite its stated policy goal of improving access to preventive and primary care, coverage status alone did not correspond to a shift toward lower-acuity emergency department presentations relative to privately insured or uninsured patients in this sample.

Government-sponsored patients accounted for the majority of emergency department visits and a substantial proportion of higher-acuity (ESI 1-2) presentations. A previous study in 2022 also reported a positive association between rising Medicaid enrollment and increased emergency department utilization at the state level [6]. These observations contrast with earlier work asserting that the Affordable Care Act coverage expansions reduced emergency department use among young adults by improving access to non-emergency care, an effect reported to be potentially stronger among patients with chronic illness [7]. While the association between insurance type and ESI level did not reach statistical significance in the present study, the absence of any apparent protective effect associated with government-sponsored coverage is a descriptive observation worth further investigation: if Medicaid meaningfully improved access to timely outpatient care, one might expect some redistribution toward lower-acuity encounters, which was not observed here.

These findings are consistent with the hypothesis that Medicaid coverage alone, independently, may be insufficient to meaningfully shift emergency department acuity. Given the non-significant result, small effect size, and single-day single-center design, this study cannot establish whether Medicaid coverage failed to reduce emergency department reliance or whether other unmeasured factors (access to primary care, appointment availability, applicable deductible costs, health literacy, or illness severity at symptom onset) played a larger role. This distinction has direct relevance to health policy discussions, since expanded coverage without parallel improvements in primary care access, reimbursement adequacy, or care coordination may not, independently, be sufficient to reduce emergency department reliance.

Notably, uninsured patients in this study did not demonstrate a disproportionate burden of high-acuity presentations, challenging the long-standing assumption that lack of insurance necessarily results in delayed, more severe presentations. This observation, similar to others in this study, is descriptive and should be confirmed in a larger sample before being generalized.

The exploratory comparison between Medicare and Medicaid subgroups suggests that at least part of the acuity observed within the broader government-sponsored category may be attributable to Medicare beneficiaries, who are expected to present with greater medical complexity related to age and comorbidity burden. This subgroup comparison was not prespecified, was not formally tabulated, and should be interpreted cautiously; it is presented to guide the design of future, higher-powered studies.

This study offers a simple, low-cost, and reproducible framework that could be scaled across institutions and extended over longer time periods, which may be useful in generating a larger evidence base for future policy-relevant analyses.

Limitations

This study has several important limitations. Most notably, data were collected over a single 24-hour period at a single institution, which substantially limits generalizability and leaves the findings susceptible to day-of-week, seasonal, and site-specific variation; a single day cannot be assumed to represent typical departmental case mix. The study was powered a priori to detect a medium effect size (Cohen's w = 0.30) with 80% power, requiring a target sample size of 167 patients; the enrolled sample of 203 patients exceeded this target and retained 88.4% power for a medium effect. The study was, however, markedly underpowered (13.2% power) to detect a small effect (w = 0.10); a sample of roughly 1,500 patients would have been needed to reliably detect an association of that magnitude. Additionally, because the achieved sample of 203 was only marginally below the 214 patients that would have been required to achieve 80% power for the specific effect size observed in this study (w = 0.265), a modestly larger single-day or multi-day sample could plausibly have yielded a statistically significant result, and the present non-significant finding should not be interpreted as strong evidence of no association for effects smaller than the prespecified medium effect size. ESI is a triage-based assessment influenced by clinical judgment and may not perfectly correlate with underlying clinical severity. Finally, the Medicare/Medicaid subgroup comparison was exploratory and post hoc, and its results require confirmation in a study specifically designed and powered for that comparison.

## Conclusions

No statistically significant association between insurance type (private, government-sponsored, or uninsured) and ESI level was identified. Government-sponsored patients accounted for the majority of emergency department visits and a substantial proportion of higher-acuity presentations. There was no descriptive evidence of a protective effect of government-sponsored coverage on presentation acuity, and uninsured patients did not show a disproportionate burden of high-acuity presentations. These preliminary, hypothesis-generating findings should be interpreted with caution given the single-day, single-center design and should be confirmed in larger, adequately powered, multicenter studies before informing policy conclusions.
